# Tendinous Tissue Adaptation to Explosive- vs. Sustained-Contraction Strength Training

**DOI:** 10.3389/fphys.2018.01170

**Published:** 2018-09-04

**Authors:** Garry J. Massey, Thomas G. Balshaw, Thomas M. Maden-Wilkinson, Neale A. Tillin, Jonathan P. Folland

**Affiliations:** ^1^Arthritis Research UK Centre for Sport, Exercise and Osteoarthritis, Loughborough University, Loughborough, United Kingdom; ^2^School of Sport, Exercise, and Health Sciences, Loughborough University, Loughborough, United Kingdom; ^3^Faculty of Health and Wellbeing, Sheffield Hallam University, Sheffield, United Kingdom; ^4^Department of Life Sciences, University of Roehampton, London, United Kingdom

**Keywords:** tendon, aponeurosis, stiffness, young’s modulus, muscle, strength training, hypertrophy

## Abstract

The effect of different strength training regimes, and in particular training utilizing brief explosive contractions, on tendinous tissue properties is poorly understood. This study compared the efficacy of 12 weeks of knee extensor explosive-contraction (ECT; *n* = 14) vs. sustained-contraction (SCT; *n* = 15) strength training vs. a non-training control (*n* = 13) to induce changes in patellar tendon and knee extensor tendon–aponeurosis stiffness and size (patellar tendon, vastus-lateralis aponeurosis, quadriceps femoris muscle) in healthy young men. Training involved 40 isometric knee extension contractions (three times/week): gradually increasing to 75% of maximum voluntary torque (MVT) before holding for 3 s (SCT), or briefly contracting as fast as possible to ∼80% MVT (ECT). Changes in patellar tendon stiffness and Young’s modulus, tendon–aponeurosis complex stiffness, as well as quadriceps femoris muscle volume, vastus-lateralis aponeurosis area and patellar tendon cross-sectional area were quantified with ultrasonography, dynamometry, and magnetic resonance imaging. ECT and SCT similarly increased patellar tendon stiffness (20% vs. 16%, both *p* < 0.05 vs. control) and Young’s modulus (22% vs. 16%, both *p* < 0.05 vs. control). Tendon–aponeurosis complex high-force stiffness increased only after SCT (21%; *p* < 0.02), while ECT resulted in greater overall elongation of the tendon–aponeurosis complex. Quadriceps muscle volume only increased after sustained-contraction training (8%; *p* = 0.001), with unclear effects of strength training on aponeurosis area. The changes in patellar tendon cross-sectional area after strength training were not appreciably different to control. Our results suggest brief high force muscle contractions can induce increased free tendon stiffness, though SCT is needed to increase tendon–aponeurosis complex stiffness and muscle hypertrophy.

## Introduction

The mechanical stiffness (resistance to deformation) of muscle tendinous tissues (aponeurosis and extramuscular free tendon) is integral to the effectiveness of these tissues to transmit skeletal muscle force to the bone and thus generate movement. Stiffer tissues may be protective in injury-related situations, for instance maintaining balance in response to mechanical perturbation (Karamanidis et al., 2008). Moreover, stiffer tendons undergo less strain in response to stress, which reduces their susceptibility to damage (Buchanan and Marsh, 2002). Likewise, stiffer tissues may limit injury risk by providing greater joint stability and by perhaps reducing the loading imposed on passive joint tissue structures (meniscus, cartilage, ligaments), (Lipps et al., 2014). A particular concern is that traumatic joint injuries predispose to degenerative disease (e.g., anterior cruciate ligament) and the increased risk of knee osteoarthritis, which contributes to a reduced quality of life (Salaffi et al., 2005). Therefore, increased tendinous tissue stiffness could have functional and clinical implications, thus identifying effective interventions to stimulate tendinous tissue adaptations is warranted.

*In vivo* tendinous tissue stiffness is typically determined from force–elongation relationships acquired by combining tissue elongation visualized via ultrasonography with estimates of tendon force during ramp isometric contractions. In response to a constant rate of increase in contractile force, elongation of the free tendon [between proximal and distal osteotendon junction’s (Kongsgaard et al., 2007; Seynnes et al., 2009)] and elongation of the distal tendon–aponeurosis complex (i.e., aponeurosis and free tendon) via the displacement of a muscle-fascicle aponeurosis intersection (Kubo et al., 2001, 2006c; Arampatzis et al., 2007) can be used to determine stiffness of both these structures. During muscle contraction the free tendon experiences tensile loading and positive longitudinal strain, whereas the radial expansion of muscle fascicles during force-generation and shortening causes the aponeurosis to also undergo transverse elongation and positive strain (Azizi and Roberts, 2009; Raiteri et al., 2016). The alternative strain behavior of the free tendon and aponeurosis may lead to differential adaptations in the separate free tendon and combined tendon–aponeurosis complex in response to training. However, very few studies have made simultaneous measurements of the mechanical properties of both structures (Kubo et al., 2006a,c, 2009), therefore the comparative changes in free tendon and tendon–aponeurosis complex stiffness after exercise training remains opaque.

The mechanical stiffness of the tendon–aponeurosis complex has been repeatedly found to increase following strength training with sustained contractions at high loads (≥2 s duration with loads of >70% maximum: Bohm et al., 2015; Wiesinger et al., 2015), e.g., 16–54% after 12–14 weeks (Kubo et al., 2001, 2006b; Arampatzis et al., 2007). Interestingly, two recent studies reported that strength training with brief explosive-contractions (<1 s) characterized by maximum/near maximum rate of force development up to a high level of force produced increases in stiffness after merely 4 (34%; Tillin et al., 2012) and 6 weeks (62%; Burgess et al., 2007) of training. These preliminary results suggest that explosive-contraction strength training (ECT) may provide a potent stimulus for increasing tendon–aponeurosis complex stiffness. Furthermore due to the brief nature of the contractions (Balshaw et al., 2016), ECT is a relatively non-fatiguing training regime that may be preferable for older adults and patient groups (e.g., mobility, limited, osteoarthritis, tendinopathy: Reid et al., 2015) and thus facilitate higher levels of adherence. However, a comprehensive longer-term investigation is required to validate the efficacy of ECT to increase tissue stiffness in comparison to more conventional sustained-contraction strength training (SCT).

Changes in tendon–aponeurosis complex and free tendon stiffness after strength training may depend upon the increase in the size of these tissues. Muscle hypertrophy is a well-recognized characteristic response to conventional strength training regimes (Folland and Williams, 2007) that is suggested to be coincident with an increase in aponeurosis size (Wakahara et al., 2015), but longitudinal changes in aponeurosis size are largely unknown. A solitary report documented a 1.9% increase in vastus lateralis aponeurosis width to accompany a 10.7% increase in quadriceps muscle size after 12 weeks of SCT (Wakahara et al., 2015). Free tendon hypertrophy after SCT has received much more attention, but the evidence remains equivocal. While some studies utilizing magnetic resonance imaging (MRI) have reported modest increases in free tendon cross-sectional area (CSA) (∼3–6%: Arampatzis et al., 2007; Kongsgaard et al., 2007; Seynnes et al., 2009; Bohm et al., 2014) that may be region specific, others found no change (Arampatzis et al., 2010; Kubo et al., 2012; Bloomquist et al., 2013). The responses of muscle, aponeurosis and tendon size to ECT are largely unknown. Given the marginal changes in free tendon size after SCT, the increases in free tendon stiffness (e.g., 15–65%: Reeves et al., 2003; Kongsgaard et al., 2007; Seynnes et al., 2009; Malliaras et al., 2013; McMahon et al., 2013) have predominantly been attributed to the nearly parallel increases in free tendon Young’s modulus (stiffness relative to tendon dimensions, i.e., material stiffness), although the changes in free tendon modulus after ECT have yet to be documented.

The aim of the present study was to comprehensively compare the mechanical and morphological adaptations of the tendinous tissues, both the patellar tendon and tendon–aponeurosis complex, to 12 weeks ECT vs. SCT vs. a non-training control group. The mechanical properties examined were patellar tendon stiffness and Young’s modulus, as well as tendon–aponeurosis complex stiffness. Morphological measures investigated were quadriceps femoris muscle volume, vastus lateralis aponeurosis area and patellar tendon CSA. As both training regimes involved high force production, we hypothesized that ECT and SCT would be similarly effective training interventions to increase tendinous tissue stiffness.

## Materials and Methods

### Participants and Ethical Approval

Forty-two young, healthy, asymptomatic, males who had not completed lower body-strength training for >18 months and were not involved in systematic physical training were randomly assigned to ECT (*n* = 14), SCT (*n* = 15) or control (CON, *n* = 13) groups. Baseline recreational physical activity level was assessed with the International Physical Activity Questionnaire (IPAQ, short format). Each participant provided written informed consent prior to completing this study, which was approved by the Loughborough University Ethical advisory committee and conformed to the principles of the Declaration of Helsinki.

### Experimental Design

Participants visited the laboratory for a familiarization session that included measurement of muscle strength and body mass to facilitate group allocation, as well as practice isometric ramp contractions. Thereafter, two duplicate laboratory measurement sessions were conducted both pre (sessions 7–10 days apart prior to the first training session) and post (2–3 and 4–6 days after the last training session). MRI scans of the thigh and knee were conducted pre (5 days prior to the start of the first training session) and post (2–3 days after the final training session) to measure knee extensor tissue size (quadriceps muscle volume, vastus lateralis aponeurosis area, patellar tendon CSA) and patellar tendon moment arm. All measurement and training sessions were performed with the same isometric apparatus and the same joint angle configuration [knee and hip angles of 115° and 126° (180° = full extension)]. Training for ECT and SCT group’s involved unilateral isometric contractions of both legs three times a week for 12 weeks (36 sessions in total), whereas CON participants attended only the measurement sessions and maintained their habitual activity. All participants were instructed to maintain their habitual physical activity and diet throughout the study, which was verified by informal questioning during post measurement. Measurement sessions involved a series of contractions of the dominant (preferred kicking) leg in the following order: maximum voluntary contraction [MVCs to establish maximum voluntary torque (MVT)]; ramp voluntary contractions of the knee extensors to establish tendinous tissue properties, and knee flexor MVCs. Knee joint torque was recorded throughout contractions. Knee flexor surface electromyography was recorded during knee flexor MVCs, as well as during knee extensor ramp contractions to account for antagonist co-activation in the estimate of tendon force in knee extensor ramp contractions. Ultrasound images of the vastus lateralis muscle and patellar tendon were recorded to assess tissue elongation during the ramp contractions in order to derive force–elongation relationships (to determine stiffness) of the distal tendon–aponeurosis complex and patellar tendon, as well as stress–strain relationships for the patellar tendon (to determine Young’s modulus). Measurement sessions were at a consistent time of day and started between 12:00 h and 19:00 h.

### Training

**FIGURE 1:**
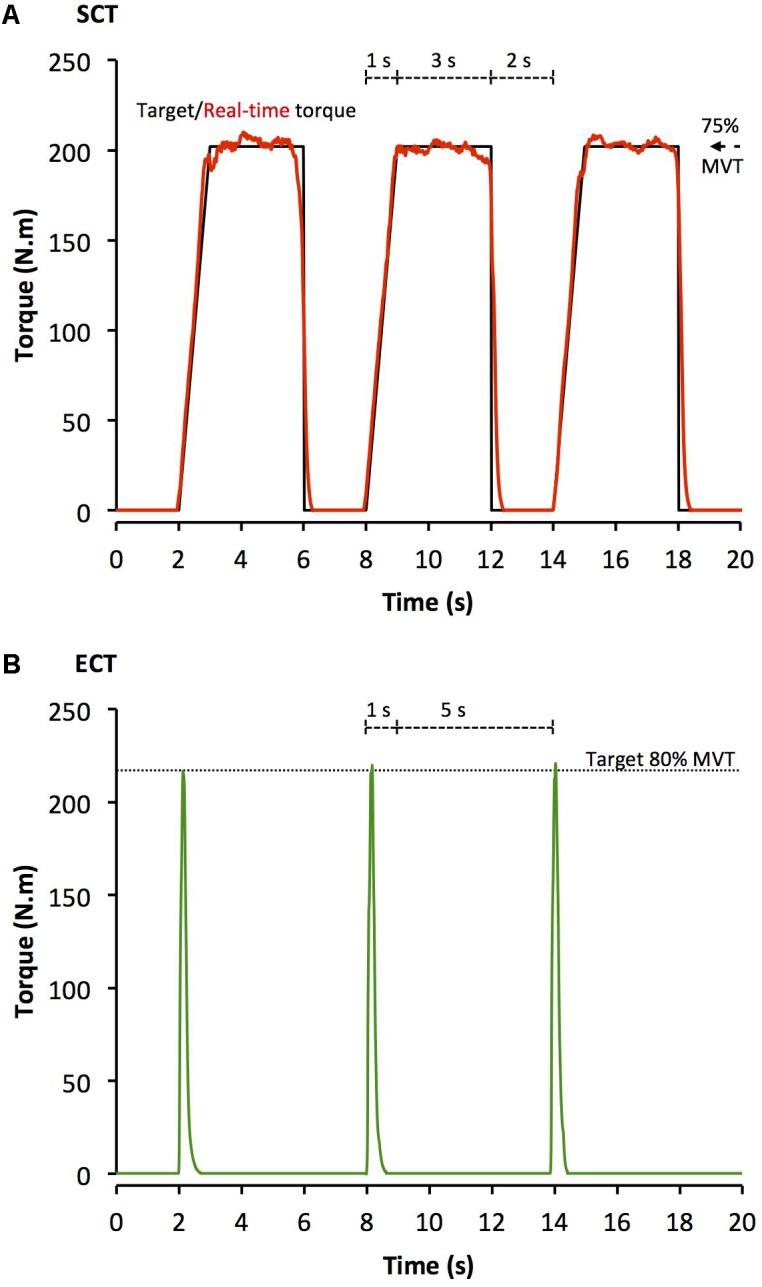
Example isometric knee extension torque–time traces performed during **(A)** sustained-contraction strength training (SCT), and **(B)** explosive-contraction strength training (ECT). MVT, maximum voluntary torque.

After a brief warm-up of sub-maximum contractions of both legs, participants completed four sets of 10 unilateral isometric knee-extensor contractions of each leg with sets alternating between legs. Each set took 60 s with 2 min between successive sets on the same leg. SCT involved sustained contractions at 75% MVT, with 2 s rest between contractions. In order to control the rate of torque development (RTD) these participants were presented with a target torque trace 2 s before every contraction and instructed to match this target, which gradually increased torque linearly from rest to 75%MVT over 1 s before holding a plateau at 75%MVT for a further 3 s (**Figure 1A**). ECT involved maximum/near maximum RTD contractions with participants instructed to perform each contraction “as fast and hard as possible” then relax for 5 s between repetitions (**Figure 1B**). When performing ECT the focus was on maximizing RTD, which means participants cannot precisely control the peak torque achieved. Therefore participants were instructed to simply achieve ∼80% MVT as quickly as possible to ensure that peak torque was at least practically equivalent to SCT. A computer monitor displayed RTD (10 ms time epoch) to provide biofeedback of explosive performance, with a cursor indicating the highest peak RTD achieved throughout the session. Participants were encouraged to achieve a higher peak RTD with each subsequent contraction. The torque–time curve was also shown: with a horizontal cursor at 80%MVT to encourage sufficiently forceful contractions, and on a sensitive scale baseline torque was highlighted in order to observe and provide feedback to participants to correctly perform the contractions by avoiding any pre-tension or countermovement. All training participants (ECT and SCT) performed three isometric knee extensor MVCs at the start of each training week in order to re-establish MVT and prescribe training torques. Torque data from each repetition of all training participants in the first session of weeks 1, 6, and 12 was analyzed and loading indices were averaged across the three sessions: SCT vs. ECT, peak loading magnitude (75 vs 81% MVT), peak loading rate (1.4 vs. 8.9% MVT.s^-1^), impulse (28212 vs. 3025 Nm.s).

### Knee Extension and Flexion Maximum Voluntary Contractions

Following a brief warm-up [3 s contractions at 50% (x3), 75% (205 x3) and 90% (x1) of perceived maximum], participants performed three to four MVCs and were instructed to either ‘push as hard as possible’ (knee extension) or ‘pull as hard as possible’ (knee flexion) for 3–5 s and rest ≥30 s. A horizontal cursor indicating the greatest torque obtained within the session was displayed for biofeedback and verbal encouragement was provided during all MVCs. The highest instantaneous torque recorded during any MVC was defined as MVT.

### Torque Measurement

Measurement and training sessions were completed in the same custom-made isometric strength-testing chair with knee and hip angles of 115° and 126° (180° = full extension), respectively. Adjustable straps were tightly fastened across the pelvis and shoulders to prevent extraneous movement. An ankle strap (35 mm width reinforced canvas webbing) was placed ∼15% of tibial length (distance from lateral malleolus to knee joint space) above the medial malleolus, and positioned perpendicular to the tibia and in series with a calibrated S-Beam strain gauge (Force Logic, Berkshire, United Kingdom). The analog force signal was amplified (×370; A50 amplifier, Force Logic, Berkshire, United Kingdom) and sampled at 2,000 Hz using an A/D converter (Micro 1401; CED, Cambridge, United Kingdom) and recorded with Spike 2 computer software (CED). In offline analysis, force signals were low-pass filtered at 500 Hz using a fourth order zero-lag Butterworth filter, gravity corrected by subtracting baseline force, and multiplied by lever length, the distance from the knee joint space to the center of the ankle strap, to calculate torque values.

### Knee Flexor Electromyography (EMG)

Surface EMG recordings over the biceps femoris and semitendinosus muscles were made with a wireless EMG system (Trigno; Delsys Inc., Boston, MA, United States) during knee flexor MVCs and knee extensor ramp contractions. Following preparation of the skin (shaving, abrading, and cleansing with alcohol) single differential Trigno standard EMG sensors (1 cm inter electrode distance; Delsys Inc., Boston, MA, United States) were attached over each muscle using adhesive interfaces. Sensors were positioned parallel to the presumed frontal plane orientation of the underlying muscle fibers at 45% of thigh length (distance from the greater trochanter to the lateral knee joint space) measured from the popliteal crease. EMG signals were amplified at source (×300; 20–450 Hz bandwidth) before further amplification (overall effective gain × 909) and sampled at 2000 Hz via the same A/D converter and computer software as the force signal, to enable data synchronization. In offline analysis, EMG signals were corrected for the 48 ms delay inherent to the Trigno EMG system. During knee flexor MVCs EMG amplitude was calculated as the root mean square (RMS) of the filtered EMG signal of the biceps femoris and semitendinosus over a 500 ms epoch at knee flexion MVT (250 ms either side of instantaneous peak torque) and averaged across the two muscles to give knee flexor EMG_MAX_.

### MRI Measurement of Muscle Tendon Unit Morphology and Moment Arm

Participants reported to the MRI scanner (1.5 T Signa HDxt, GE) having not engaged in strenuous activity in the prior 36 h, and were instructed to arrive in a relaxed state having eaten and drunk normally, and sat quietly for 15 min prior to their MRI scans. T1-weighted MR images of the dominant leg (thigh and knee) were acquired in the supine position at a knee angle of 163° due to constraints in knee coil size (180° = full extension) and analyzed using OsiriX software (Version 6.0, Pixmeo, Geneva, Switzerland). Using a receiver 8-channel whole body coil, axial images (image matrix 512 × 512, field of view 260 mm × 260 mm, pixel size 0.508 mm × 0.508 mm, slice thickness 5 mm, inter-slice gap 0 mm) were acquired from the anterior superior iliac spine to the knee joint space in two overlapping blocks. Oil filled capsules placed on the lateral side of the thigh allowed alignment of the blocks during analysis. The anatomical CSA of each of the four constituent quadriceps femoris muscles (vastus lateralis, vastus intermedius, vastus medialis, and rectus femoris) was manually outlined in every third image (i.e., every 1.5 cm) starting from the most proximal image in which the muscle was visible. A cubic spline curve was fitted to the plot of anatomical CSA vs. femur length for each constituent muscle, and the muscle volume calculated as the area under the spline curve (GraphPad Prism 6, GraphPad Software, Inc.) Total quadriceps femoris muscle volume was given by the sum of the constituent muscle volumes.

**FIGURE 2:**
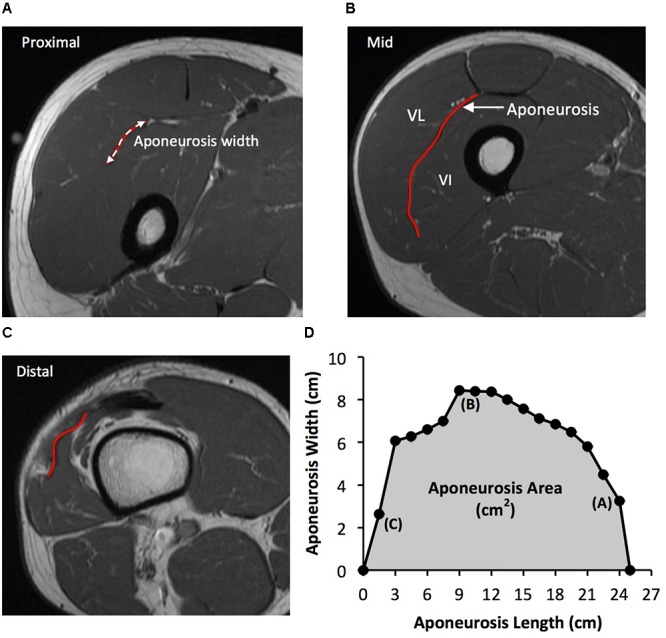
Example axial magnetic resonance images: **(A)** most proximal, **(B)** middle, and **(C)** most distal, showing the transverse length of the vastus lateralis (VL) deep aponeurosis which was traced manually in order to measure aponeurosis width. **(D)** A cubic spline curve was fitted through the aponeurosis width data points measured at 1.5 cm intervals from the most proximal and distal image where the aponeurosis was visible (aponeurosis length) and the area under the curve was defined as vastus lateralis aponeurosis area.

As previously described (Wakahara et al., 2015), the deep aponeurosis of the vastus lateralis muscle was defined as the visible dark black segment between the vastus lateralis and vastus intermedius muscles in the axial thigh MRI images (**Figure 2**). The transverse length (cm) of the black segment was defined as vastus lateralis aponeurosis width, and was traced manually on every third image (i.e., every 1.5 cm), starting in the most distal image where the aponeurosis was visible. From the images analyzed, the measures of aponeurosis width were plotted against femur length. A cubic spline curve was fitted to the plot of VL aponeurosis width vs. femur length and the vastus lateralis aponeurosis area was calculated as the area under the spline curve (**Figure 2**).

**FIGURE 3:**
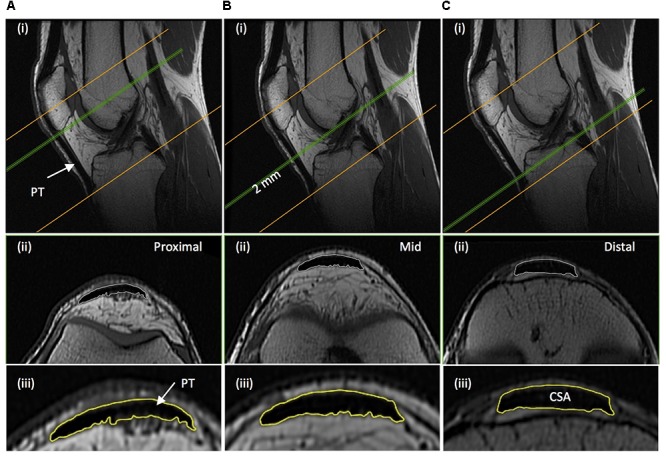
Example magnetic resonance images of the knee: **(A)** proximal; just distal to the apex of the patella, **(B)** mid-length; 50% distance between the patella-tibia attachments, and **(C)** distal; just proximal to the tendon tibial insertion. (i) Sagittal images show the position along the tendon length, of where the example axial images shown (ii) were acquired perpendicular to the tendon line of action. (iii) The perimeter of the patellar tendon (PT) was manually traced to determined PT cross-sectional area (CSA), with the average of the measures from each contiguous 2 mm image spanning tendon length being defined as mean patellar tendon CSA.

Immediately after thigh imaging, a lower extremity knee coil was used to acquire axial (image matrix 512 × 512, field of view 160 mm × 160 mm, pixel size 0.313 mm × 0.313 mm, slice thickness 2 mm, inter-slice gap 0 mm) and sagittal images (image matrix 512 × 512, field of view 160 mm × 160 mm, pixel size 0.313 mm × 0.313 mm, slice thickness 2 mm, inter-slice gap = 0 mm) of the knee joint. Contiguous axial images spanned patellar tendon length, which prior to analysis were reconstructed with an orientation perpendicular to the patellar tendon via the mutli-plane view feature of OsiriX. Images spanned from 2 cm superior to the patella apex to 2 cm inferior to the tendon tibial insertion. Patellar tendon CSA was measured on each contiguous image along the tendon’s length (first image where the patellar was no longer visible to the last image before the tibial insertion). Images, viewed in grayscale, were sharpened and the perimeter manually outlined (**Figure 3**). Mean tendon CSA (mm^2^) was defined by the average of all measured analyzed images. Patellar tendon moment arm length was estimated from sagittal plane images, as the perpendicular distance from the patellar tendon to the midpoint of the distance between the tibio-femoral contact points in the lateral and medial femoral condyles (Blazevich et al., 2009; Seynnes et al., 2009).

### Ramp Contractions for Determination of Tendinous Tissue Stiffness

**FIGURE 4:**
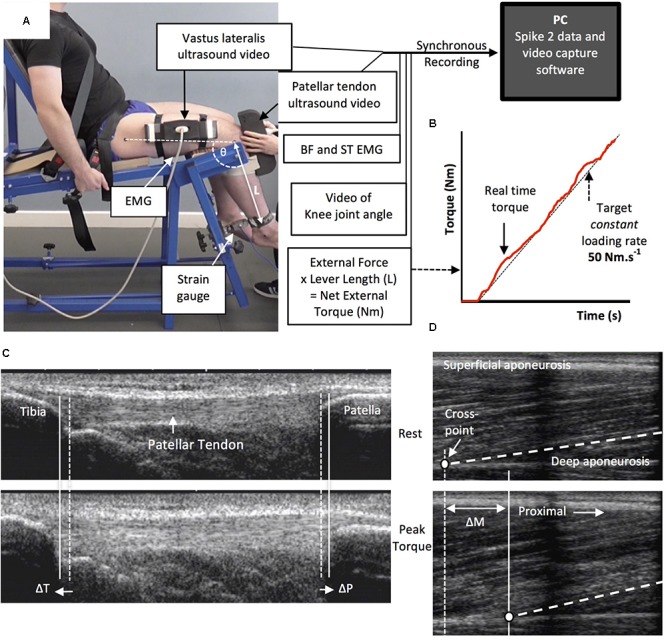
The experimental set-up and ultrasound images during the ramp contractions. Participants were tightly fastened to a rigid isometric strength-testing chair with resting knee and hip angles of 115° and 126°, respectively **(A)**. Unilateral knee extension torque, video of the knee joint angle, antagonist muscle [biceps femoris (BF), semitendinosus (ST)] surface electromyography (EMG) and ultrasound video images were synchronously recorded during constant-loading rate isometric ramp knee extensor contractions (example in **B**). Ultrasound images are of the patellar tendon **(C)** and vastus lateralis muscle **(D)** at rest (top) and at peak ramp torque (bottom) and indicate the measurement of patellar tendon (tibia-patella apex displacement, ΔT + ΔP) and tendon–aponeurosis complex (vastus lateralis muscle fascicle-deep aponeurosis cross point proximal displacement, ΔM) elongation.

Tendinous tissue stiffness was derived from synchronous recordings of torque and tissue elongation (corrected for passive tissue displacement via video recording of knee joint changes; see below) during isometric knee extension ramp contractions (experimental set-up: **Figure 4**). Participants completed two sub-maximum practice ramp contractions prior to five maximum attempts with 90 s of rest between contractions. Prior to each ramp contraction participants were shown a target torque–time trace on a computer monitor that increased at a constant gradient (50 Nm.s^-1^ loading rate) from zero up to MVT. They were instructed to match the target trace as closely as possible for as long as possible (i.e., up to MVT), and then relax promptly. Real-time torque was displayed over the target rising torque–time trace for feedback. The preceding knee extensor MVCs and sub-maximum contractions were considered sufficient to elicit tissue preconditioning (Seynnes et al., 2014). The three most suitable ramp contractions, according to highest peak torque, the closeness to the target loading rate, as well the clarity of the ultrasound images of both the patellar tendon and vastus lateralis muscle (clearly visible osteotendon attachments and fascicle-aponeurosis intersection), were analyzed and measurements averaged across these three contractions.

### Measurement of Tendinous Tissue Elongation

Two ultrasound machines and a camera were interfaced with the computer collecting torque data in Spike 2, and video images were synchronously recorded with torque (and EMG) using Spike 2 video capture at 25 Hz. Video images were captured to obtain tissue (tendon–aponeurosis and patellar tendon) and knee joint displacements during ramp contractions, which were measured in off-line analysis by tracking specific anatomical landmarks frame-by-frame in public domain semi-automatic video analysis software: Tracker, version 4.86 (www.physlets.org/tracker/).

An ultrasound linear array probe (60 mm, B-mode, 7.5 MHz scanning frequency, 39 Hz sampling frequency, Toshiba Power Vision 6000, SSA-370A) was fitted into a custom made high-density foam cast that was strapped to the lateral aspect of the thigh with the mid-point of the probe positioned at ∼50% thigh length. The probe was aligned so the fascicles inserting into the vastus lateralis muscle deep aponeurosis could be visualized at rest and during contraction. An echo absorptive marker (multiple layers of transpore medical tape) was placed beneath the ultrasound probe to provide a reference for any probe movement over the skin. Vastus lateralis muscle fascicle deep aponeurosis cross-point displacement relative to the skin marker provided a measure of distal tendon–aponeurosis complex elongation (**Figure 4**). To enable correction of aponeurosis displacement due to joint angle changes during ramp contractions, individual ratios of aponeurosis displacement relative to joint angular displacement (mm/°) were obtained from passive movements (i.e., plotting the aponeurosis displacement-knee joint angle relationship). The mean ± standard deviation for this ratio was 0.37 ± 0.09 mm/°. Passive movements were conducted prior to the ramp contractions. Participants were instructed to completely relax as their knee was moved through 90°–130°. During passive movements and ramp contractions, knee joint angle (angle between visible markers placed on the greater trochanter, lateral knee joint space and lateral malleolus) was derived from sagittal plane video recorded using a camera mounted on a tripod positioned (1.5 m) perpendicular to the strength-testing chair. During ramp contractions knee angle changes were 3.1 ± 1.2°.

A second ultrasound linear array probe (92 mm EUP-L53L, B-mode, 10 MHz scanning frequency, 32 Hz sampling frequency; Hitachi EUB-8500) was fitted into a custom made high-density foam cast that was held firmly over the anterior aspect of the knee with the probe aligned longitudinal to the patellar tendon such that the patella apex and insertion of the posterior tendon fibers at the tibia could be visualized at rest and throughout the contraction. Patellar tendon elongation was determined by the longitudinal displacement of both the patella apex and the tendon tibial insertion (**Figure 4**). Under passive conditions, patellar tendon elongation was deemed negligible.

### Calculation of Patellar Tendon Force

Patellar tendon force was calculated by dividing total knee extensor torque by the patellar tendon moment arm length. Direct measures of moment arm were acquired at rest from MRI images as indicated above (MRI measurement). Due to constraints in the size of the knee coil, sagittal images were acquired in an extended knee position (∼163°: 180° = full extension). Moment arm length for any specific knee angle measured at rest or during ramp contraction was estimated from previously published data fitted with a quadratic function (Kellis and Baltzopoulos, 1999) scaled to each participant’s measured moment arm length at 163°. Total knee extensor torque was given by summing external net knee extension torque and the estimated knee flexor co-contraction torque. Antagonist knee flexor torque was estimated by expressing the average knee flexor EMG amplitude (RMS 50 ms moving window) during ramp contractions relative to the knee flexor EMG_MAX_, and then multiplying by the knee flexor MVT (assuming a linear relationship between EMG amplitude and torque). During analysis, torque and EMG amplitude were down sampled to 25 Hz to match the ultrasound video recording.

### Calculation of Tendinous Tissue Stiffness and Patellar Tendon Young’s Modulus

For each of the three best ramp contractions analyzed, both patellar tendon and distal tendon–aponeurosis complex (corrected for passive tissue displacement due to knee joint angle displacement) and during elongation contraction were separately plotted against total tendon force (corrected for antagonist force). Patellar tendon and tendon–aponeurosis complex and force–elongation plots were fitted with a second-order polynomial. To standardize the tendon force level, both pre and post-training, tendon–aponeurosis complex and patellar tendon stiffness for each individual was calculated as the slope of the respective force–elongation curve over an absolute tendon force range that equated to 70–80% of pre-training MVT. 70–80% pre-training MVT corresponded to the highest common torque range that all participants could individually achieve during pre-training measurements sessions Patellar tendon Young’s modulus was calculated for each individual as the slope of the stress–strain curve derived over a stress range that corresponded to 70–80% of pre-training MVT. Stiffness/modulus measures derived over the highest attainable force/stress range are recommended and deemed suitably reliable (Hansen et al., 2006; Kösters et al., 2014; Seynnes et al., 2014). Tendon stress was obtained by dividing tendon force by mean patellar tendon CSA. Patellar tendon strain was the percentage tendon displacement relative to the resting tendon length. Resting patellar tendon length was defined as the distance between the patella apex and tibial insertion as measured prior to the ramp contractions. The measures of patellar tendon and tendon–aponeurosis complex stiffness, and the patellar tendon modulus derived from each of the three analyzed ramps were averaged to give a representative value for each individual.

### Statistical Analysis

The reproducibility of measurements (all muscle and tendinous tissue variables) over the 12-week intervention period was calculated for CON (pre vs. post) as within-participant coefficient of variation [CVw, %; (SD/mean) × 100]. Muscle and tendon variables measured during the duplicate laboratory sessions were averaged to produce criterion pre and post values for statistical analysis. Data are reported as mean ± standard deviation (SD). Statistical significance tests were conducted using SPSS Version 20.0 (IBM Corp., Armonk, NY, United States), and significance was accepted at *p* < 0.05. 0.05 < *p* < 0.1 was considered a tendency. One-way analysis of variance (ANOVA) tests were conducted on all pre-training variables to determine whether baseline differences existed between groups. The primary comparison of training effects involved between group comparisons to the intervention, and assessment of repeated measures analysis of variance [ANCOVA; group (ECT vs. SCT vs. CON) × time (pre vs. post)] with corresponding pre-training values used as covariates. When group × time interaction effects displayed *p* < 0.05, least significant difference (LSD) *post hoc* pairwise comparisons [with Holm–Bonferroni adjustment applied to the *p*-values (LSD_HB_)] of absolute changes (pre to post) between groups (i.e., ECT vs. SCT, ECT vs. CON, SCT vs. CON) were performed to delineate specific between-group differences. In addition to the between group comparisons, secondary within-group changes (absolute values) were evaluated with paired *t*-tests. Effect size (ES: specifically Hedges g, incorporating correction for small sample bias; Lakens, 2013) was calculated for between-group comparisons and within group changes.

## Results

### Group Characteristics at Baseline

At baseline, no differences (*p* ≥ 0.579) were observed between groups for age (ECT 25 ± 2; SCT 25 ± 2; CON 25 ± 3 years), height (ECT 174 ± 7; SCT 175 ± 8; CON 176 ± 6 cm), body mass (ECT 71 ± 10; SCT 70 ± 8; CON 72 ± 7 kg) or habitual physical activity level (ECT 1971 ± 1077; SCT 2084 ± 1256; CON 2179 ± 1588 metabolic equivalent minutes per week). Likewise, there were no differences in MVT (*p* = 0.304), tendon–aponeurosis complex stiffness (*p* = 0.328), patellar tendon stiffness (*p* = 0.215), Young’s modulus (*p* = 0.184), quadriceps muscle volume (*p* = 0.508), and vastus lateralis aponeurosis area (*p* = 0.815), though a tendency existed for patellar tendon mean CSA (*p* = 0.073).

### Reproducibility of Measurements

The reproducibility of pre and post measures for the CON group over the 12-week intervention period was excellent for MVT (CVw 2.9%) and tendon–aponeurosis complex stiffness (3.9%), and very good for patellar tendon stiffness (7.2%) and Young’s modulus (6.8%). Excellent reproducibility was also observed for quadriceps muscle volume (1.7%), vastus lateralis aponeurosis area (2.7%) and patellar tendon mean CSA (2.9%).

### Strength and Muscle–Tendon Morphology (**Tables 1**, **2** and **Figure 5**)

**Table 1:** Strength, muscle–tendon unit size, patellar tendon moment arm, and patellar tendon and tendon–aponeurosis complex mechanical properties.

	Explosive-contraction strength		Sustained-contraction strength		Non-training		Two-way ANCOVA
	training (ECT)		training (SCT)		control (CON)		Group ×
							time (*p* value)
			
	Pre	Post	Pre	Post	Pre	Post	
**Strength and morphology**					
Maximum voluntary torque (MVT), Nm	234 ± 27	273 ± 36^∗∗∗^_L_	237 49	293 ± 47^∗∗∗^_L_	255 ± 50	256 ± 58	<0.001
Quadriceps muscle volume, cm^3^	1778 ± 244	1827 ± 277	1820 ± 273	1967 ± 316^∗∗∗^_S_	1897 ± 282	1909 ± 271	0.018
Vastus lateralis aponeurosis area, cm^2^	137.1 ± 16.4	143.1 ± 15.2^∼^_S_	136.3 ± 26.1	144.3 ± 21.2^∗^_S_	138.8 ± 13.7	140.5 ± 15.7	0.242
Patellar tendon mean CSA, mm^2^	98.7 ± 10.0	95.9 ± 8.3^∗^_S_	97.3 ± 12.9	97.7 ± 13.0	106.5 ± 9.0	103.6 ± 10.7^∗^_S_	0.129
Patellar tendon length, mm	47.5 ± 5.7	47.2 ± 5.7	45.4 ± 5.5	45.1 ± 5.5	47.1 ± 5.7	46.6 ± 6.8	0.829
Patellar tendon moment arm, mm	40.6 ± 2.4	40.7 ± 2.3	42.4 ± 2.9	42.5 ± 2.9	41.2 ± 2.9	41.3 ± 2.9	0.902
				
**Patellar tendon properties**		
Elongation at 80% pre-MVT, mm	3.17 ± 0.52	2.82 ± 0.42^∗∗^_M_	3.23 ± 0.54	3.07 ± 0.64	3.12 ± 0.62	3.02 ± 0.63	0.270
Stiffness, N.mm^-1^	2605 ± 446	3122 ± 632^∗∗^_L_	2835 ± 444	3239 ± 575^∗^_M_	2534 ± 501	2569 ± 413	0.018
Strain at 80% pre-MVT, %	6.8 ± 1.7	6.0 ± 1.1^∗∗^_M_	7.2 ± 1.4	6.9 ± 1.7	6.6 ± 1.1	6.4 ± 1.1	0.093
Young’s modulus, GPa	1.23 ± 0.18	1.49 ± 0.27^∗∗∗^_L_	1.32 ± 0.27	1.51 ± 0.36^∗^_M_	1.14 ± 0.27	1.16 ± 0.20	0.012
			
**Tendon–aponeurosis complex properties**			
Elongation at 80% pre-MVT, mm	15.0 ± 2.6	17.4 ± 2.2 ^∗∗^_L_	16.9 ± 4.6	16.4 ± 5.3	16.3 ± 5.7	16.6 ± 4.4	0.020
Stiffness, N.mm^-1^	592 ± 118	595 ± 101	560 ± 177	687 ± 285^∗∗^_M_	507 ± 130	511 ± 116	0.007

*Data are mean ± SD. ECT, *n* = 13; SCT, *n* = 15 (size and strength), *n* = 14/15 (tendon–aponeurosis complex/patellar tendon); CON, *n* = 13 (size and strength) and *n* = 13/12 (tendon–aponeurosis/patellar tendon). ^∗∗∗^Different to pre, *p* ≤ 0.001, ^∗∗^*p* < 0.01, ^∗^*p* < 0.05. ∼0.05 < *p* < 0.1. Within-group effect size: S, small (0.2–0.5); M, moderate (>0.5–0.8), L, large (>0.8)*.

**Table 2:** Summary of within-group changes and between-group differences from pre to post training in strength, muscle–tendon unit morphology and tendinous tissue stiffness indices.

	Within-group changes			Between-group differences
	Explosive-contraction	Sustained-contraction	Non-training	
	strength training (ECT)	strength training (SCT)	control (CON)	
**Strength and morphology**
Maximum voluntary torque (MVT), Nm	↑ +17%	↑ +24%	↔	ECT and SCT↑ > CON
Quadriceps muscle volume, cm^3^	↔	↑ +8%	↔	SCT↑ > CON
Vastus lateralis aponeurosis area, cm^2^	↔	↑ +7%	↔	-
Patellar tendon mean CSA, mm^2^	↓ -3%	↔	↓ -3%	-
				
**Tendinous tissue stiffness indices**				
*Patellar tendon*				
Elongation at 80% pre-MVT, mm	↓ -10%	↔	↔	-
Strain at 80% pre-MVT, %	↓ -11%	↔	↔	-
Stiffness, N.mm^-1^	↑ +20%	↑ +16%	↔	ECT and SCT↑ > CON
Young’s modulus, GPa	↑ +22%	↑ +16%	↔	ECT and SCT↑ > CON
				
*Tendon–aponeurosis complex*				
Elongation at 80% pre-MVT, mm	↑ +17%	↔	↔	ECT↑ > SCT
Stiffness, N.mm^-1^	↔	↑ +21%	↔	SCT↑ > ECT and CON

*The directions of the group changes are shown by ↑ or ↓ with the percentage change in the group mean also shown. Non-significant within-/between group changes are indicated by ↔/-*.

**FIGURE 5:**
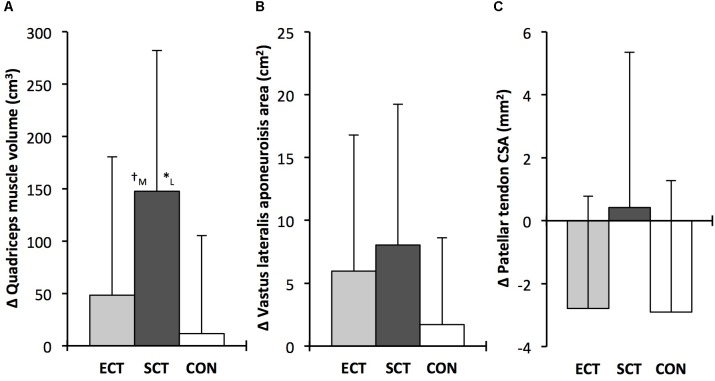
Pre to post absolute changes (Δ) in **(A)** quadriceps femoris muscle volume **(B)** vastus lateralis aponeurosis area and **(C)** patellar tendon mean cross-sectional area (CSA) in response to explosive-contraction (ECT, *n* = 13) or sustained-contraction strength training (SCT, *n* = 14) interventions and in a non-training control group (CON, *n* = 13). Symbols indicate between-group differences: ^∗^SCT vs. CON, *p*<0.05; ^†^ECT vs. SCT, trend 0.05 < *p* < 0.09. Letter denotes effect size magnitude: M, moderate (0.5–0.8); L, large (>0.8). Data are group mean ± SD.

Considering within-group changes, MVT increased after ECT (paired *t*-test *p* < 0.001, ES = 1.15) and SCT (*p* < 0.001, ES = 1.11) but not following CON (*p* = 0.868, ES = 0.01). Between group comparisons showed the absolute increase in MVT was greater than CON for both ECT (LSD_HB_ *p* < 0.001, ES = 1.90) and SCT (LSD_HB_ *p* < 0.001, ES = 2.64), and 45% larger after SCT than ECT (LSD_HB_ *p* = 0.032, ES = 0.75).

Quadriceps muscle volume increased after SCT (paired *t*-test *p* = 0.001, ES = 0.47) but not following ECT (*p* = 0.195, ES = 0.17) or CON (*p* = 0.661, ES = 0.04). There was a group × time effect for quadriceps muscle volume (**Table 1**), with the absolute change (**Figure 5A**) after SCT being greater than CON (LSD_HB_ *p* = 0.021, ES = 1.12), and a tendency to be different to ECT (*p* = 0.074, ES = 0.72). Absolute changes in quadriceps muscle volume after ECT were not greater than CON (LSD_HB_ *p* = 0.479, ES = 0.31).

Vastus lateralis aponeurosis area increased after SCT (paired *t*-test *p* = 0.015, ES = 0.32), and also tended to increase after ECT (*p* = 0.060, ES = 0.35), while remaining unchanged in CON (*p* = 0.408, ES = 0.11). However, there was no group × time effect (**Table 1** and **Figure 5B**).

Patellar tendon mean CSA showed a small decrease in CON (paired *t*-test *p* = 0.028, ES = 0.27), and after ECT (*p* = 0.012, ES = 0.29), but was unchanged following SCT (*p* = 0.746, ES = 0.03). However, there was no group × time effect (**Table 1** and **Figure 5C**).

### Tendinous Tissue Mechanical Properties (**Tables 1**, **2**)

**FIGURE 6:**
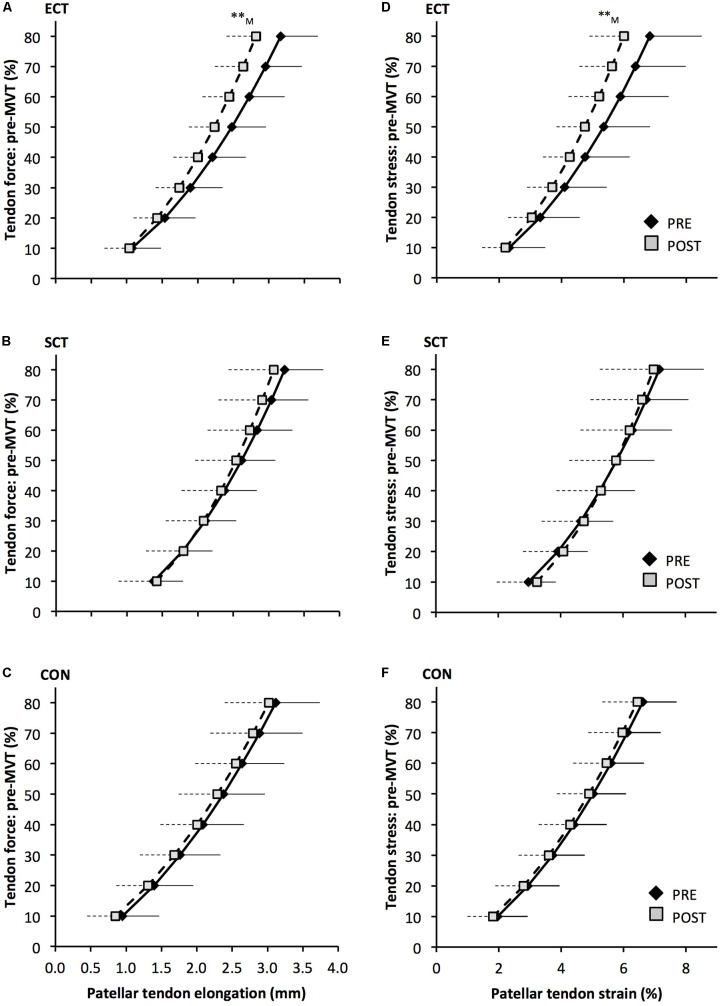
Patellar tendon force- elongation **(A–C)** and stress–strain **(D–F)** relationships pre (black diamonds) and post (gray squares) 12 weeks of explosive-contraction [ECT, *n* = 13 **(A,D)**] or sustained-contraction [SCT, *n* = 15 **(B,E)**] strength training interventions and in an untrained control group [CON, *n* = 12 **(C,F)**]. Data are group mean ± SD. Data points are plotted at the elongation or strain corresponding to tendon force or stress at 10% increments of pre-training maximum voluntary torque (MVT). Symbols indicate within-group difference ^∗∗^*p*<0.01. Letter denotes effect size magnitude: M, medium (0.5–0.8).

Patellar tendon elongation at 80% pre-training MVT was less after ECT (paired *t*-test *p* = 0.011, ES = 0.75, but was unchanged after SCT (*p* = 0.246, ES = 0.24) or CON (*p* = 0.331, ES = 0.15), (**Figure 6**), and no group × time effect was observed (**Table 1**). Patellar tendon strain (relative elongation) at 80% pre-training MVT was also less after ECT (paired *t*-test *p* = 0.010, ES = 0.54), but was unchanged after SCT (*p* = 0.542, ES = 0.11) or CON (*p* = 0.263, ES = 0.15), (**Figure 6**), and there was no group × time effect (**Table 1**).

**FIGURE 7:**
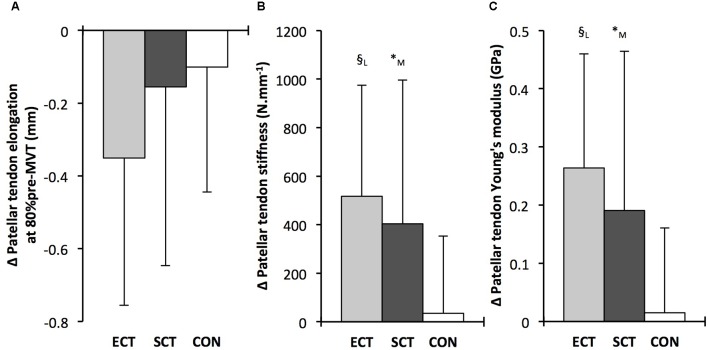
Pre to post absolute changes (Δ) in **(A)** Patellar tendon elongation at 80% of pre-training maximum voluntary torque (MVT), **(B)** patellar tendon stiffness, **(C)** patellar tendon Young’s modulus, in response to explosive-contraction (ECT, *n* = 13) or sustained-contraction (SCT, *n* = 15) strength training interventions and in a non-training control group (CON, *n* = 12). Symbols indicate between-group differences: ^§^ ECT vs. CON *p* < 0.05; ^∗^SCT vs. CON, *p* < 0.05; Letter denotes effect size magnitude: M, moderate (>0.5–0.8); L, large (>0.8). Data are mean ± SD.

Patellar tendon stiffness increased after both ECT (paired *t*-test *p* = 0.002, ES = 0.88) and SCT (*p* = 0.019, ES = 0.74), but was unchanged in CON (*p* = 0.711, ES = 0.07). There was a group × time effect (**Table 1**), and absolute changes (**Figure 7**) in both ECT (LSD_HB_ *p* = 0.030, ES = 1.18) and SCT (LSD_HB_ *p* = 0.034, ES = 0.73) were greater than CON. ECT and SCT had a similar effect on patellar tendon stiffness (LSD_HB_ *p* = 0.500, ES = 0.29).

Patellar tendon Young’s modulus increased after ECT (paired *t*-test *p* = 0.004, ES = 1.05), and SCT (*p* = 0.017, ES = 0.57), and was unchanged in CON (*p* = 0.637, ES = 0.05), resulting in a group × time effect (**Table 1**). Absolute changes (**Figure 7**) were greater in both ECT (LSD_HB_ *p* = 0.012, ES = 1.38) and SCT (LSD_HB_ *p* = 0.042, ES = 0.75) than CON. Positive effects of ECT and SCT on tendon Young’s modulus were similar (LSD_HB_ *p* = 0.830, ES = 0.21).

**FIGURE 8:**
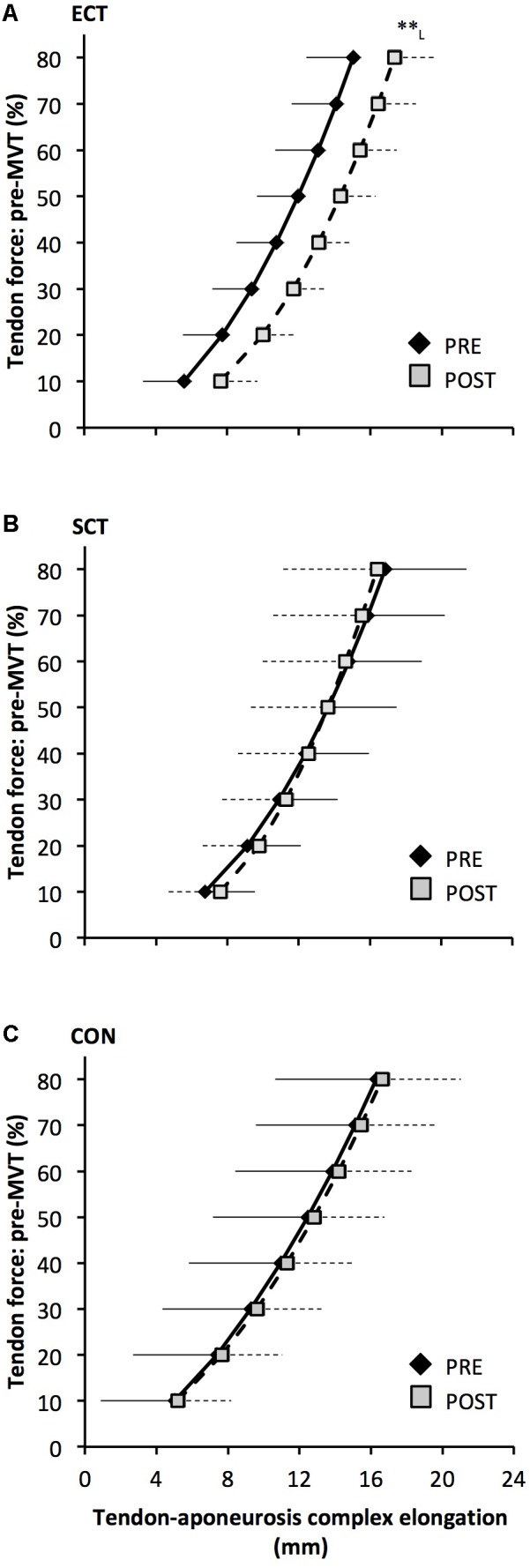
Tendon force-tendon–aponeurosis complex elongation relationships pre (black diamonds) and post (gray squares) 12 weeks explosive-contraction [ECT, *n* = 13 **(A)**] or sustained-contraction [SCT, *n* = 15 **(B)**] strength training interventions and in a non-training control group [CON, *n* = 13 **(C)**]. Data are group mean ± SD. Data points are plotted at the elongation corresponding to tendon forces at 10% increments of pre-training maximum voluntary torque (MVT). Within-group effect, tendon–aponeurosis complex elongation at 80% pre-training MVT, post different to pre ^∗∗^*p* < 0.01. Letter denotes effect size magnitude: L, large (>0.8).

**FIGURE 9:**
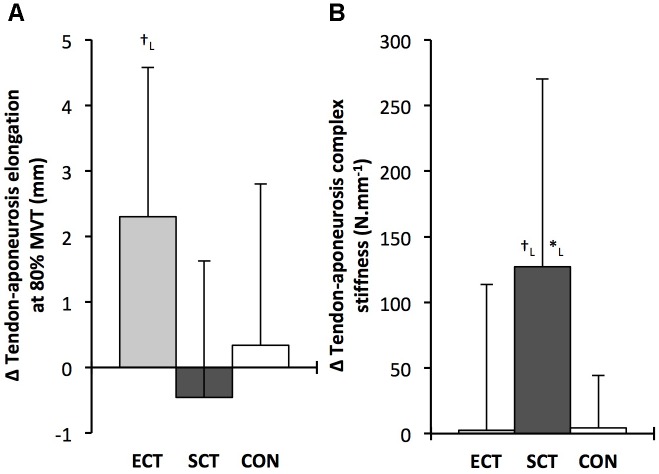
Pre to post absolute changes (Δ) in **(A)** tendon–aponeurosis complex elongation at 80% pre-training MVT and **(B)** tendon–aponeurosis complex stiffness, in response to explosive-contraction (ECT, *n* = 13) or sustained-contraction (SCT, *n* = 14) strength training interventions and in a non-training control group (CON, *n* = 13). Symbols indicate between-group differences: ^∗^SCT vs. CON, *p* < 0.05; ^†^ECT vs. SCT *p* < 0.05. Letter denotes effect size magnitude: L, large (>0.8). Data are mean ± SD.

Tendon–aponeurosis complex elongation at 80% pre-training MVT increased after ECT (paired *t*-test *p* = 0.003, ES = 0.89) but was unchanged after SCT (*p* = 0.428, ES = 0.09) and CON (*p* = 0.637, ES = 0.06), (**Figure 8**). There was a group × time effect (**Table 1**), with increases in ECT being greater than SCT (LSD_HB_ *p* = 0.021, ES = 1.23) and tended to be greater than CON (LSD_HB_ *p* = 0.098, ES = 0.80) (**Figure 9**).

Tendon–aponeurosis complex stiffness increased after SCT (paired *t*-test *p* = 0.005, ES = 0.50) but was unchanged after ECT (*p* = 0.938, ES = 0.02) and CON (*p* = 0.695, ES = 0.03,), with a group × time effect (**Table 1**). Absolute changes in tendon–aponeurosis complex stiffness (**Figure 9**) following SCT were greater than ECT (LSD_HB_ *p* = 0.015, ES = 0.94) and CON (LSD_HB_ *p* = 0.016, ES = 1.12), while ECT vs. CON changes were alike (LSD_HB_ *p* = 0.846 ES = 0.02).

## Discussion

The present randomized controlled study compared the efficacy of 12 weeks of explosive- (ECT) vs. sustained- (SCT) contraction strength training to increase patellar tendon stiffness and Young’s modulus, knee extensor tendon–aponeurosis complex stiffness as well as elicit tissue (muscle, aponeurosis, free tendon) hypertrophy. ECT and SCT similarly increased patellar tendon stiffness and modulus (20 and 22% vs. 16 and 16%), whereas only SCT increased tendon–aponeurosis complex stiffness (21%), and quadriceps muscle volume (8%). There was a marginal effect of SCT on aponeurosis area (within-group increase, but no between group differences), while patellar tendon hypertrophy was not clearly apparent after either SCT or ECT.

Sustained-contraction training increased high-force free tendon stiffness, as has been commonly reported in response to strength training regimes utilizing sustained (>2 s) high force (>70% maximum) dynamic and/or isometric muscle contractions (e.g., Seynnes et al., 2009; Malliaras et al., 2013; McMahon et al., 2013). A more original finding was the increase in free tendon stiffness after ECT, as this had not been investigated in previous studies (Burgess et al., 2007; Tillin et al., 2012). Intriguingly, ECT (+20%) was similarly effective as SCT (+16%) for stimulating increases in free tendon high-force stiffness, and both increased by more than CON. The greater patellar tendon stiffness after ECT and SCT can be explained by the parallel increase in patellar tendon Young’s modulus in response to training. This adaptation to SCT is consistent with multiple previous studies (Seynnes et al., 2009; Malliaras et al., 2013; McMahon et al., 2013) although the similar effect of ECT on free tendon Young’s modulus we have observed has not been investigated before. Our findings support the view that the changes in free tendon Young’s modulus is the primary mechanism for the increased in tendon stiffness during the initial months of strength training (Wiesinger et al., 2015). Increased Young’s modulus after SCT and ECT may be due to changes to the patellar tendon intrinsic collagenous structure and/or biochemical composition, e.g., increased collagen content, cross-link density, fibril size (Buchanan and Marsh, 2002; Kjaer et al., 2015). At present evidence for specific alterations in free tendon intrinsic structure/composition after strength training in healthy individuals are lacking, and therefore further investigations to uncover the mechanism(s) for the increases in Young’s modulus are required.

The similar increases in patellar tendon Young’s modulus after ECT and SCT may be attributable to their similar loading magnitude (%MVT). It is recognized that *in vitro* mechanotransduction responses of tenocytes (resident tendon cells responsible for extracellular matrix remodeling) are highly dependent on strain magnitude (Lavagnino et al., 2008) as reflected by *in vivo* studies showing increased free tendon stiffness and modulus only after high vs. low force strength training (Kongsgaard et al., 2007; Arampatzis et al., 2010). The similar changes to free tendon Young’s modulus after ECT and SCT despite the previously documented (Balshaw et al., 2016) differences in time related loading parameters with these training regimes (loading rate, ECT 6-fold > SCT; loading duration SCT 13-fold > ECT), strongly suggests that loading magnitude, irrespective of duration or rate, is the primary mechanostimulatory parameter for the free tendon.

In the present study, the increases in patellar tendon stiffness in ECT and SCT were independent of free tendon hypertrophy. Whilst it is curious there was a small within-group decrease in mean patellar tendon CSA in CON, this possible negative bias in post-training measures had only a small effect size (0.27). Moreover, the primary between group comparisons, that is the most robust indicator of training effects in comparison to CON, revealed no between group differences. Several earlier studies have similarly reported no change in free tendon CSA after a comparable period of SCT (Arampatzis et al., 2010; Kubo et al., 2012; Bloomquist et al., 2013). However, others have reported small increases in free tendon CSA following similar SCT regimes (∼3–6%: Arampatzis et al., 2007; Kongsgaard et al., 2007; Seynnes et al., 2009; Bohm et al., 2014). With regards to our patellar tendon mean CSA data it is unlikely that our measurements simply failed to detect a change. Pre and post free tendon CSA analysis was performed by a single investigator blinded to the group allocation, and involved precise measurements of tendon CSA along the full length of the tendon from high resolution MRI (2 mm slice thickness, pixel size 0.313 mm × 0.313 mm), with excellent reproducibility even over the duration of the intervention (∼3% pre–post CVw in CON). It is possible the magnitude of tendon hypertrophy after relatively short-term resistance training is small, and on the borderline of what can be detected. Importantly, however, we recently found no evidence for free tendon hypertrophy in long-term (4 years) resistance trained men, despite their substantially greater muscle volume (56%) and strength (58%) compared to untrained controls (Massey et al., 2017). Based on those findings and the current results it seems unlikely that high-load resistance training causes tendon hypertrophy even after months and years of training.

Moreover, the lack of free tendon hypertrophy after strength training in the current study is consistent with some evidence that resistance exercise/training may not noticeably stimulate increased *in vivo* collagen protein synthesis. For instance, an acute bout of high load dynamic knee extensor contractions (3 × 10 repetitions, 70% 1 repetition maximum) had no effect on patellar tendon collagen type I messenger RNA expression 24 h post exercise (Sullivan et al., 2009). Also, 12 weeks of isoinertial squat training failed to increase the concentration of procollagen type 1 *N*-propeptide (biomarker of collagen synthesis) in patellar tendon peritendinous tissue [Bloomquist et al., 2013; this study also observed no change in patellar tendon CSA (via MRI)]. Contrarily there is some evidence that mechanical loading of free tendon tissue can induce an increased collagen synthesis (Miller et al., 2005) although it is not a consistent finding (Dideriksen et al., 2013). Therefore mechanical loading *in vivo* may not necessarily stimulate a sufficiently robust induction of the appropriate biochemical response needed to elicit free tendon hypertrophy.

In contrast to the free tendon, the tendon–aponeurosis complex stiffness measured at high force levels (i.e., 70–80% pre-training MVT) increased only after SCT, but not ECT. The increased tendon–aponeurosis complex high force stiffness after SCT is consistent with previous findings (Kubo et al., 2001; Arampatzis et al., 2007, 2010; Bohm et al., 2014) and the greater increase after SCT than ECT may be attributable to the substantially longer loading duration in SCT. Previous work has shown greater increases in tendon–aponeurosis complex stiffness after strength training with long vs. short duration contractions (Kubo et al., 2001; Arampatzis et al., 2007). The absence of change in tendon–aponeurosis complex stiffness for ECT in the current study contrasts with earlier studies examining the triceps surae (Burgess et al., 2007) and knee extensors (Tillin et al., 2012). It is possible that our results diverge from Burgess et al. (2007), because an increase in free tendon stiffness as we have observed after ECT, may be of greater consequence to the triceps surae tendon–aponeurosis complex, as the Achilles tendon accounts for a larger proportion of the triceps surae tendon–aponeurosis complex stiffness (Farcy et al., 2013). Tillin et al. (2012) trained their participants at a longer muscle length (knee joint angle 85° vs. 115° in the current study), which has been shown to result in greater increases in knee extensor tendon–aponeurosis which has been shown to result in greater increases in knee extensor tendon–aponeurosis complex stiffness (Kubo et al., 2006b) in accordance with high force development in conditions of higher tissue strain magnitude (McMahon et al., 2013), and this could explain their contrasting findings of increased knee extensor tendon–aponeurosis complex stiffness.

An interesting observation was that the force–elongation relationship post ECT was actually shifted to the right (greater elongation at specific forces). The increase in elongation in response to the same high force after ECT was greater than after SCT and tended to be greater than the CON group. The rightward shift in the force–elongation curves after ECT appears to result from a change in elongation at the initial level (10%MVT), that persists throughout the rise in tendon force, as after 10%MVT the gradients of the force–elongation relationships pre–post ECT are equivalent. Consistent with our data, there is some evidence that sprint trained athletes (who inherently utilize explosive contractions) display greater knee extensor tendon–aponeurosis complex elongation at the lowest levels of force (<20%MVT), with resultantly greater elongation throughout the measured force range (Kubo et al., 2000, 2011). It is possible that a reduction in low force tendon aponeurosis complex stiffness (i.e., 0–10% MVT) after ECT with no changes at higher forces indicates changes in tissue collagenous structure/composition that specifically influence the lower region of the force–elongation relationship. In contrast, whilst SCT increased high force stiffness there was no clear leftward shift in the force–elongation curve. Indeed, some previous studies have concordantly reported an increase in high force tendon–aponeurosis complex stiffness, along with no apparent effect on the elongation at lower force levels (Kubo et al., 2001, 2010). These results perhaps imply that SCT may induce tissue collagenous structure/composition changes that specifically impact the high stiffness region of the force–elongation relationship (e.g., collagen cross-links: Kjaer et al., 2015). Further work is needed to fervently elucidate whether force level specific changes in stiffness are likely to occur with different interventions, and identify any possible mechanistic basis for this supposition.

Collectively our findings show that in comparison to a control intervention patellar tendon stiffness but not tendon–aponeurosis complex stiffness increased after ECT, whereas SCT increased both patellar tendon and tendon–aponeurosis complex stiffness, indicating a differential adaptive response of these tendinous tissues according to the training regime. The contrasting patellar tendon and tendon–aponeurosis complex stiffness changes after ECT demonstrates the independence of these adaptations. The simple observation that only a small proportion of tendon–aponeurosis complex elongation is due to the patellar tendon elongation (19%) further highlights the distinction of these measures. From our study we cannot discount a contribution of the quadriceps tendon and vastus lateralis extramuscular tendon to tendon–aponeurosis complex stiffness because the fascicle-aponeurosis intersection displacement reflects elongation in all tendinous tissues distal to the tracked point (Stafilidis et al., 2005). However, from our data and previous measures of vastus lateralis myotendinous junction and aponeurosis elongation (Stafilidis et al., 2005), the muscle aponeurosis apparently comprises the most influential component of tendon–aponeurosis elongation and stiffness. The tendon–aponeurosis complex stiffness changes after SCT could reflect adaptations (material properties and/or size) of the aponeurosis component of the tendon–aponeurosis complex, and there was some indication of increased aponeurosis area after SCT (+7% within-group change, but insufficient for a between group effect), that could conceivably have contributed to the increased tendon–aponeurosis complex stiffness after SCT. Aponeurosis hypertrophy is thought to be necessary to provide an enlarged attachment area for an increased muscle CSA (Wakahara et al., 2015), thus our finding is consistent with the similar hypertrophic response of the quadriceps femoris muscle (+8%) after SCT and not ECT (or CON). The muscle hypertrophic response to SCT but not ECT is most likely a consequence of the greater total loading duration with SCT. Following bouts of isoinertial knee extensions with equivalent load, a greater total loading duration was associated with increased acute amplitude of muscle myofibrillar protein synthesis (Burd et al., 2012). Therefore, the limited total loading duration in ECT is perhaps an insufficient stimulus for the necessary muscle protein synthesis, and likely accounts for the lack of muscle hypertrophy in response to this training modality. Although it should be recognized that overall muscle volume is a relatively gross measure that may not capture regional remodeling or hypertrophy within specific regions of the muscle according to localized mechanical stimuli.

A potential limitation of our study concerns the methodology for determining tendon–aponeurosis mechanical properties, even though it has been used very extensively (Kubo et al., 2001, 2006b, 2009; Bojsen-Møller et al., 2005; Tillin et al., 2012). In addition to the patellar tendon, which we have assessed, the contribution of other intermediary tendinous tissues (i.e., quadriceps and vastus lateralis tendon), to tendon–aponeurosis complex elongation appears relatively small (Stafilidis et al., 2005), but has limited attention. The measurement of tendon–aponeurosis complex elongation could also be influenced by the active state of muscle fibers in parallel with the aponeurosis. Aponeurosis stiffness is considered muscle-activation dependent as muscle fibers anchor the aponeurosis during contraction (Lieber et al., 2000), and is also modulated by muscle deformation during contraction (Azizi and Roberts, 2009) as well as the relative force distribution along the length of the aponeurosis (Zuurbier et al., 1994). Training-induced changes in muscle morphology and architecture, as well as neural recruitment strategy along the muscle length, may have influenced muscle–aponeurosis interaction and thus aponeurosis behavior during contraction, conceivably confounding the interpretation of differences in tendon–aponeurosis stiffness pre–post intervention. However, at present we are not aware of a better technique for investigating the mechanical behavior of the tendon–aponeurosis complex.

## Conclusion

In conclusion, ECT was equally effective as SCT for stimulating an increase in patellar tendon stiffness and Young’s modulus, demonstrating that in order to induce free tendon adaptation, strength training need only involve brief, high force muscle contractions. However, brief high force muscle contractions are not solely sufficient to stimulate muscle and aponeurosis adaptations as only SCT increased tendon–aponeurosis complex stiffness, muscle size, and aponeurosis size, while ECT was ineffective. Thus our results suggest muscle–aponeurosis adaptations are specific to the loading regime and sensitive to loading duration.

## Author Contributions

GM, TB, TM-W, NT, and JF conceived and designed the study. GM, TB, and TM-W performed the experiments. GM, TB, TM-W, and NT analyzed the data. GM and JF interpreted the data and drafted the manuscript. TB, TM-W, and NT critically evaluated the manuscript. All authors are responsible for the final content of the manuscript.

## Conflict of Interest Statement

The authors declare that the research was conducted in the absence of any commercial or financial relationships that could be construed as a potential conflict of interest.
